# Electrochemical Lignin Oxidation Reaction on CuO: *In Situ* Spectroelectrochemical Point of View

**DOI:** 10.1021/acs.langmuir.5c02744

**Published:** 2025-08-15

**Authors:** André H. B. Dourado, Matheus Santos, Ana P. de Lima Batista, Antonio G. S. de Oliveira-Filho, Antonio A. S. Curvelo, Hamilton Varela

**Affiliations:** † São Carlos Institute of Chemistry, University of São Paulo, Av. Trab. Sancarlense, 400, São Carlos 13566-590, Brazil; § Departamento de Química, Grupo Computacional de Catálise e Espectroscopia (GCCE), 201361 Universidade Federal de São Carlos (UFSCar), São Carlos, SP 13565-905, Brazil

## Abstract

Lignins are macromolecules present in biomass tissues, the third most prevalent component. This compound is frequently used as a thermoelectric fuel generating cheap energy; however, it burns the most abundant renewable source of aromatic structures in the world. For obtaining useful low-molecular weight compounds, depolymerization needs to be considered. One way of accomplishing this is the lignin oxidation reaction, which can be catalytic or noncatalytic. The noncatalytic type uses hard chemicals, temperature, and pressure and can present lower selectivity, further oxidizing monoaromatic products. The use of catalysts, especially electrocatalysts, makes the conditions mild and increases the selectivity; however, the reaction pathway followed is still under debate. It is difficult to find proposals in the literature that consider the heterogeneous catalyst–lignin interaction, so in this work, we applied *in situ* spectroelectrochemical techniques in the infrared region to check this, using commercial CuO catalyst powder as the catalyst and sugar cane lignin in 2.0 mol L^–1^ KOH. The spectra were registered in external reflection–absorption configuration and deconvoluted, and the intensities and position of the bands were analyzed. For a deeper understanding of the chemical adsorption, the first step of lignin electro-oxidation, computational simulations were performed for a model lignin molecule with C_γ_ as the C atom closest to the CuO surface. The C_α_–C_β_ breaking linkages could be followed, and some improved mechanistic steps were proposed.

## Graphical abstract

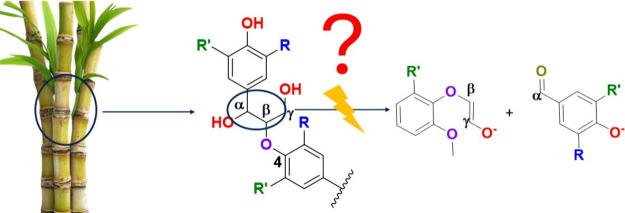

## Introduction

Lignins are phenolic macromolecules and some of the main constituents of lignocellulose biomass, along with cellulose and hemicelluloses. Consisting of three main aromatic alcohol precursors, *p*-coumaryl alcohol (H), coniferyl alcohol (G), and sinapyl alcohol (S), lignins present a complex chemical structure (Scheme 1).^1^ Due to its in-plant polymerization process and the varying availability of each precursor, interunit linkages in lignin can be either the carbon–carbon type, such as β–β or β–5, or the ether type, β-O-4 being the most abundant one (Scheme 1).^1^ As a result, its structure and composition are highly variable.^2,3^ Lignin is the most abundant renewable phenolic material in nature and is largely released during the pulping process. Its abundance and renewability make it an attractive feedstock to produce phenolic compounds.^4,5^ However, unlike cellulose and hemicelluloses, the selective depolymerization of lignins remains a major challenge.

**Scheme 1:**
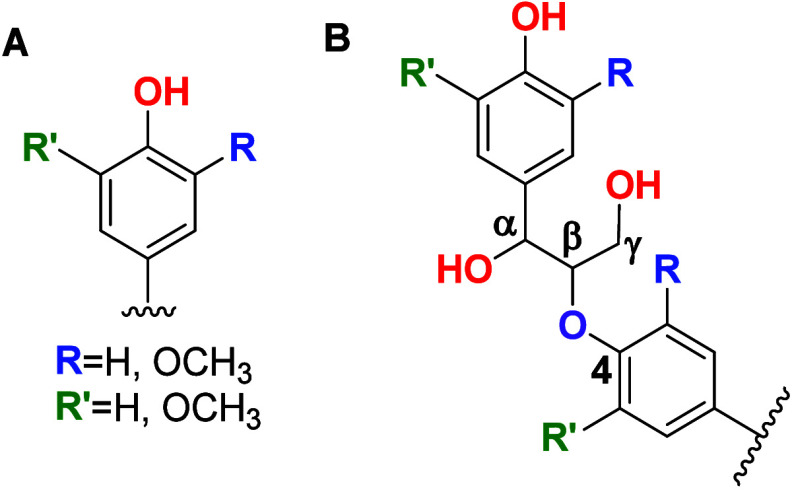
(A) Representations of the Lignin Monomers’ Basic Structure[Fn sch1-fn1] and (B) Representation of the β-O-4 Linkage between the Monomers, the Most Prevalent in the Lignin Used in This Work^6^

a
*p*-Coumaryl alcohol (H) corresponds to R = R′ = H, coniferyl alcohol (G) to R ≠ R′, and sinapyl alcohol (S) to R = R′ = OCH_3_.

Different approaches for lignin depolymerizations have been reported, from chemical and biological processes.^7,8^ Among the chemical methods, redox chemistry has shown great results. Both reduction and oxidation reactions were widely explored as a method for lignin depolymerization and valorization, each with its advantages and drawbacks.^9–12^ Although reduction is more selective and can avoid the recondensation of lignin, in the oxidative method, careful control of the reaction conditions can avoid overoxidation and enhance the depolymerization process, even cleaving carbon–carbon interunit linkages.^13,14^ Nonetheless, while in the reductive method lignin-derived products are less oxygenated, presenting saturated side chains and possibly being hydrogenated to produce cyclohexanols, the products from oxidation are more functionalized phenols, such as aldehydes, ketones, and acids.^12,13^

Several oxidants have been proposed for the oxidative depolymerization of lignins into monomers, among which nitrobenzene has resulted in the highest yields. However, nitrobenzene is reduced into toxic products that are difficult to separate from the oxidized lignin monomers, and O_2_ has been used with or without a metal catalyst as an alternative.^15,16^ Nonetheless, the use of a metal-based catalyst can improve the yield of the oxidation. A wide range of metal salts, mostly homogeneous catalysts, and oxides, mostly heterogeneous, have already been employed in lignin oxidation; recent reports show the use of copper oxide affords greater efficiency, leading to monophenol yields that can surpass the theoretical maximum.^14^

As depolymerization can occur through redox processes, electrocatalysis presents a viable method for facilitating this transformation.^17,18^ Therefore, reduction of lignin can, once more, be found in the electrochemical literature,^18,19^ and as presented previously for thermocatalysis, the main products for the depolymerization are saturated compounds, meanly due to the presence of adsorbed hydrogen, which can react to insaturations by nonredox Langmuir–Hinshelwood^20^ or Eley–Rideal mechanisms, simultaneous to reductive bond breaking. The oxidative path, on the other hand, can maintain aromaticity and generate interesting chemicals.^6,21^

Products from thermochemical and electrochemical oxidation reactions are similar, both approaches producing aromatic aldehydes with high selectivity, accompanied by ketones and acids.^6,22^ Recently, it was shown that under alkaline conditions and with CuO, the mechanism of reaction for the oxidative depolymerization of lignin must be the same under either electro- or thermocatalysis.^6^

The oxidation mechanism of lignin has been examined in various studies, and multiple pathways have been proposed for producing aldehydes from lignin. These mechanisms are summarized in Scheme 2. Aldehydes, such as vanillin and syringaldehyde, can be produced from alkaline hydrolysis of lignins without the addition of an oxidant (yellow path in Scheme 2). Even if the focus was not to elucidate an oxidation mechanism, Hibbert proposed that the formation of vanillin from lignosulfonate in an alkaline solution happens through a retro aldol step.^23^

**Scheme 2:**
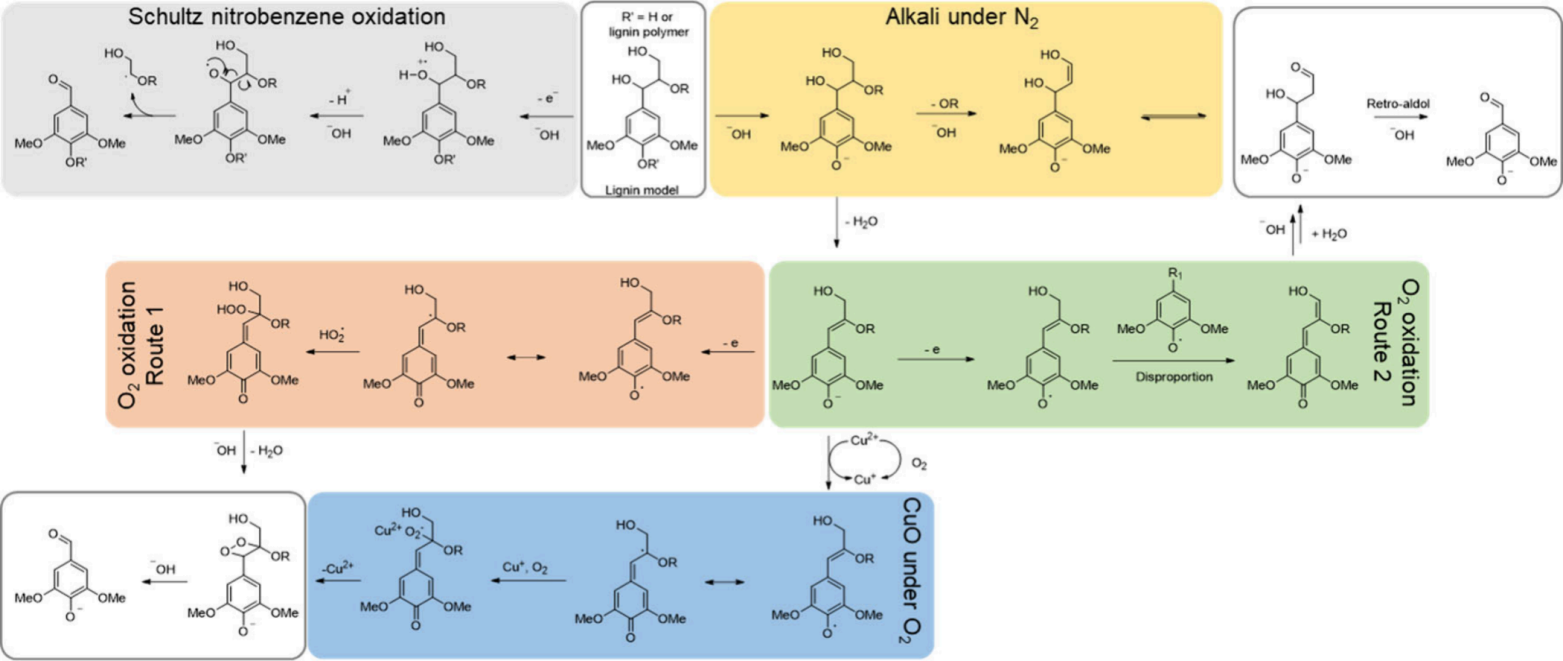
Summary of Mechanisms Proposed for the Lignin Oxidation Reaction

The nitrobenzene oxidation mechanism (gray path in Scheme 2) was proposed by Schultz. It begins with nitrobenzene abstracting an electron from the hydroxyl group at the α position.^24^ However, this pathway cannot be expanded to explain the formation of aceto derivatives.

When the oxidant is oxygen, two different mechanistic pathways have been proposed, both beginning with the abstraction of an electron from the phenolate anion and the formation of a phenoxy radical. From this radical, both proposals converge at the methylene quinone following different routes to reach it (salmon and green paths in Scheme 2). As shown in the salmon-colored path (Scheme 2), the methylene quinone can be formed through resonance within the aromatic ring, resulting in an unpaired electron on the β carbon of the side chain. The reactive radical, in the presence of an active oxygen molecule, undergoes radical coupling, followed by the formation of a dioxetane cycle intermediate. The cleavage of this four-membered ring results in C_α_–C_β_ bond cleavage, producing the aldehyde.^22^ This pathway has also been proposed when the reaction is performed with oxygen in the presence of copper oxide as the catalyst (blue path in Scheme 2).^14^

The second proposed mechanism for the formation of the methylene quinone intermediate, presented in the green pathway (Scheme 2), suggests the abstraction of a second electron from C_γ_, which could happen by the action of a second radical molecule, which abstracts a hydrogen from the γ position. The structure formed can be converted into an aldehyde at the γ position by tautomerism. A subsequent retro aldol step would then result in the formation of the desired α-aldehydes.^22^ In this scenario, a metal catalyst such as CuO could facilitate the process by promoting the electron abstraction of the phenolate anion.^22^

Additionally, the oxidation reaction in the presence of CuO and absence of O_2_ could follow a path similar to the green route presented for the reaction under oxygen. CuO would abstract an electron from the phenolate anion. However, due to the absence of another oxidant, the reduced form of CuO could not be oxidized to its active form, and a catalytic cycle would not be established.^14^

As electro- and thermocatalysis have been shown to share the mechanism for lignin oxidation using CuO,^6^ electrochemistry can be further employed as a tool for the investigation of the oxidation mechanism. The electrochemical cell allows the use of coupled techniques, such as *in situ* spectroelectrochemistry in the infrared region, which was used to elucidate the mechanism of many reactions.^25–28^ In the literature, the lignin electrochemical oxidation reaction (LEOR) was only reported for nonaqueous solvents, using model molecules instead of real lignins. Furthermore, the mechanism for C_α_–C_β_ bond cleavage was not previously explored.^29^ Thus, we proposed the use of *in situ* electrochemical infrared reflection–absorption spectroscopy (IRRAS) for the investigation of LEOR on the CuO catalyst using the macromolecule itself in alkaline media. Even with some previous work using online product detection^30^ and some electronic *in situ* spectroscopy,^31^ IRRAS has not been presented in the literature, to the best of our knowledge, especially using the whole macromolecule as the electroactive species. Since adsorption represents the initial phase of heterogeneous catalysis, computational simulations of the spectra were performed to provide information about this stage of the lignin–surface interaction.

## Experimental Section

### Chemicals

All solutions were prepared using ultrapure water (Milli-Q system). The lignin was obtained as described in the next section and, after that, dissolved in 2 mol L^–1^ KOH (Sigma-Aldrich). The final lignin concentration was 3 g L^–1^. The CuO was acquired from Synth.

### Lignin Extraction

Lignin was isolated from sugar cane bagasse fiber using a dioxane/water solution, following a previously reported method.^6^ This process, conducted under mild conditions, preserves native lignin structures, including a high content of ether linkages (e.g., β-O-4) and low levels of condensation. These structural features facilitate the depolymerization of lignin into monophenols.^6,32^

### Experimental Methods

All spectroelectrochemical experiments were performed on a three-electrode cell, the working one being a Au disk one (*d* = 10 mm), the auxiliary one being a platinum plate that surrounded the working electrode, and the reference one being an encapsulated H_2_ bubble into a Luggin capillary, against which all potentials in this work are referenced. The working electrode, after being modified by catalyst deposition, was pressed at the center of a ZnSe semispheric window.

The working electrode was modified by drop casting 10 μL of a 3.4 mg μL^–1^ CuO suspension. The electrode was then dried with a thermal blower, followed by binder casting, with 10 μL of a 0.5% (m/m) Nafion solution.

The electrolyte was 3 g of L^–1^ lignin in 2 mol L^–1^ KOH. It was purged with argon for 30 min before the measurements. The gas flow was kept at the head space during the measurements.

The potential perturbation program was controlled by an Autolab potentiostat/galvanostat (PGSTAT320N), and the potential was disturbed between 1.00 and 2.00 V_RHE_ using steps of 50 mV. The spectroscopic data were accumulated using a VERTEX 70v Bruker spectrometer with an LN-MCT Mid detector without polarization. The measurements were taken by accumulation of 128 interferograms at every potential step.

For the electrochemical *I–E* profile, a regular three-electrode cell was used employing the same electrodes and potentiostat. The potential was disturbed in a triangular program between 0.6 and 2.10 V_RHE_ at 10 mV s^–1^. For this experiment, we used a lignin concentration of 10 g L^–1^, so it could be comparable with the literature.^6,33^ The concentration was lower for the spectroscopic measurements, so data acquisition was possible.

All measurements were repeated to ensure that the data could be reproduced.

### Theoretical Methods

Using computational chemistry to study a complex structure like lignin is highly challenging. Therefore, the β-O-4 dimer (e.g., guaiacylglycerol-β-guaiacyl ether) was selected as a lignin model, as it represents 50–80% of the interunit linkages^34–37^ and aligns with the sugar cane lignin employed in this study (Figure 1A).

**Figure 1:**
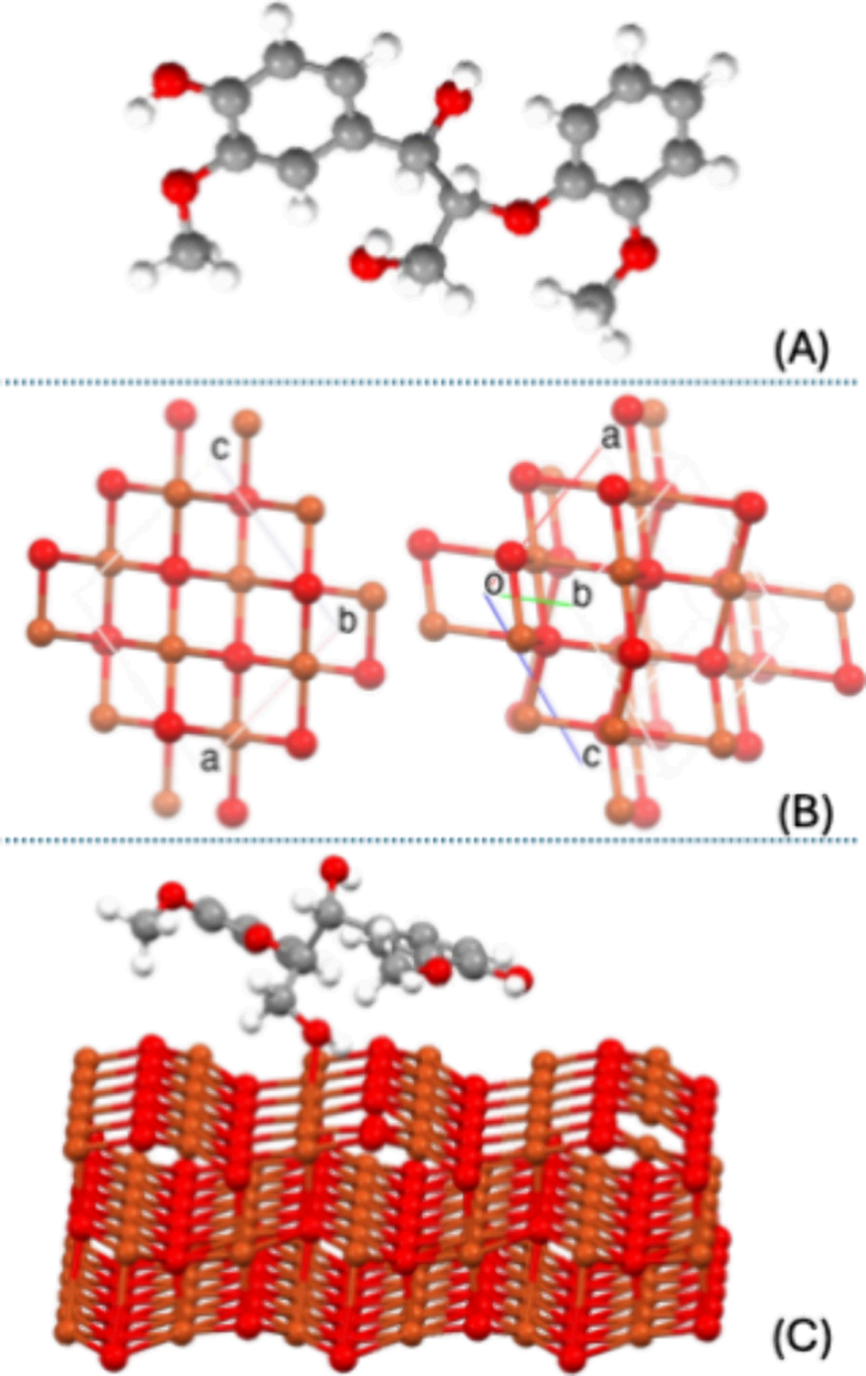
Schematic of the model structures used in this work: (A) lignin, (B) CuO unit cell in two different views, and (C) lignin/CuO (111) slab model. Hydrogen atoms are colored white, carbon atoms gray, oxygen atoms red, and copper atoms orange.

Experiments and calculations have both demonstrated that CuO (111) is the most stable surface of copper oxide, and the surface most easily observed in experiments.^38,39^ The unit cell geometry has been created by the Atomic Simulation Environment (ASE),^40,41^ as shown in Figure 1B. The CuO unit cell parameters are *a* = 4.6855 Å, *b* = 3.42169 Å, and *c* = 5.1302 Å, and the Cu–O bond length is 1.959 Å.^42,43^ The clean CuO (111) slab is a Cu_108_O_108_ stoichiometric system with three CuO layers separated by a vacuum space of 10 Å to reduce the interactions between the slab and its periodic replicas.

To explore the lignin/CuO (111) interaction and obtain the optimized structure, only the first CuO layer and the adsorbate were free to relax in all directions (Figure 1C). The minimization procedure was carried out using the EquiformerV2-31M-S2EF-OC20-All+MD machine learning model from FAIRChem Open Catalyst.^44,45^ All computations were made possible by using different modules of ASE.

Once the minimized lignin/CuO (111) geometry was obtained, Langevin (constant particle number, volume, and temperature) molecular dynamics were conducted for generating a trajectory. The trajectory was generated using a time step of 0.5 fs and a friction coefficient of 0.01 fs^–1^. The temperature and simulation time were at 300 K and 5 ps, respectively. Starting with the final trajectory, TRAVIS (Trajectory Analyzer and Visualizer)^46–48^ was be used to produce a power spectrum (vibrational density of states (VDOS)) for the whole a system, lignin on CuO (111), and for a selected pair of atoms. Hence, the resulting spectra were related to the IRRA spectra obtained for the initial stages of lignin adsorption on CuO (111).

## Results and Discussion

Before initiating the spectroelectrochemical investigation, the electrochemical profile was recorded using cyclic voltammetry, as shown in Figure 2. In Figure 2, it is notable, in the inset, that around 1.10 V_RHE_ there is a slight current increment, which is intensified around 1.45 V_RHE_. In a previous work, we have shown that between 1.0 and 1.40 V_RHE_ the current increment is observable, but it is still below the Tafel region, which should start at potentials greater than 1.40 V_RHE_.^6^ In the product analysis performed in that work, it was observed that below the Tafel region, a preference for C_β_–C_γ_ bond breaking was observed, and ketones were the major products, but at an extremely low activity. Regardless, in the Tafel region, a major selectivity for the C_α_–C_β_ bond is observed, with faradaic efficiencies close to 25%. In that work, product analysis has shown that for potentials more positive than 1.80 V_RHE_ the monoaromatic generation decreases under 10%, but the reasons are not fully comprehended. The presence of the OER needs to be considered, and it can contribute to the degradation of these monoaromatics due to the generation of highly oxidative species that are known intermediates of the oxygen evolution reaction, like H_2_O_2_.

**Figure 2:**
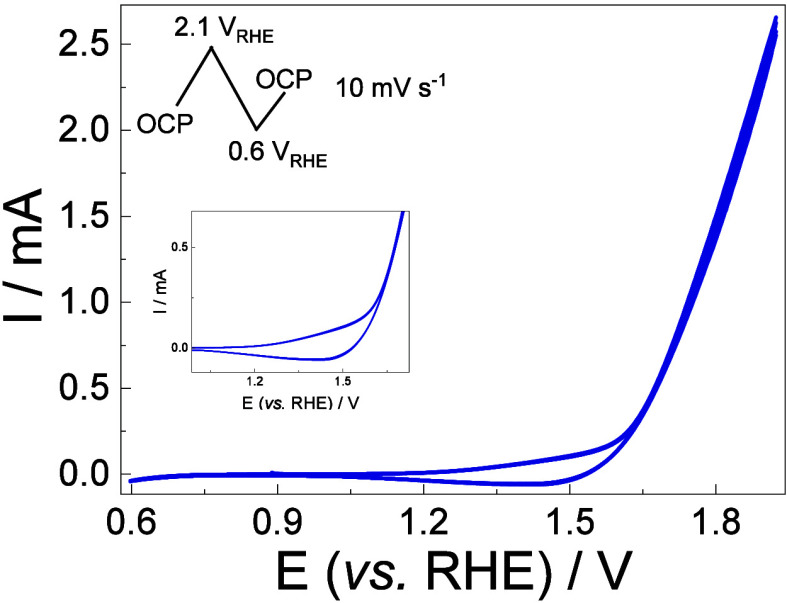
*I–E* profile for the CuO electrocatalyst applied to the lignin electrochemical oxidation reaction. Electrolyte: 10 g L^–1^ lignin and 2 mol L^–1^ KOH.

For mechanistic insights into the LEOR, which could also be extrapolated to the lignin thermochemical oxidation reaction,^6^ we decided to apply *in situ* FTIR spectroscopy for the IRRAS configuration. Examples of the resulting spectra are shown in Figure 3A.

**Figure 3:**
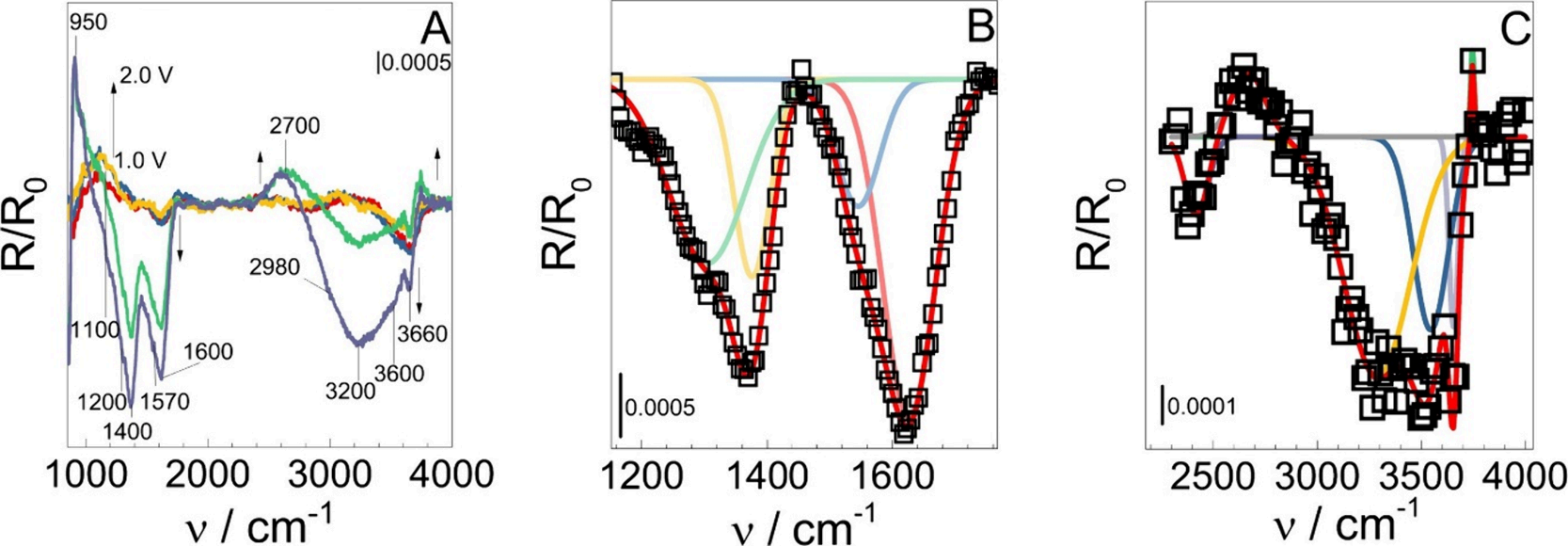
(A) *In situ* IRRA spectra obtained at different potentials during the LEOR. Arrows indicate the spectral changes due to the potential increment, and the black lines indicate the band positions (in inverse centimeters). The bands were deconvoluted, as shown in panel B for lower wavenumbers and panel C for higher.

The spectra recorded at different potentials (Figure 3A) show negative bands, which should be related to the generation of moieties or their attraction to the electrode surface. The positive bands should be related to consumption or repulsion of other moieties. Many bands are non-Gaussian-shaped, which can be related to the superposition of different oscillators, so they needed to be deconvoluted, as one can see in panels B and C of Figure 3.

In previous studies,^6^ it was shown that the lignin used presented the three monomers, units H, G, and S, and that the most frequent linkage between them was the β-O-4 linkage (Scheme 1). For unit H, R and R′ are H atoms. For unit G, R is OCH_3_ while R′ is H. For unit S, R and R′ are OCH_3_ substituents.

Taking the structures in Scheme 1, the bands obtained after deconvolution were around 950 cm^–1^, related to a polarized C–C stretching,^49^ like C_α_–C_β_. The one at 1100 cm^–1^ can also be related to C_β_–C_γ_ stretching.^50^ At 1200 cm^–1^, the band can be attributed to the C–O stretching of ether,^51^ as a combined oscillator composed of ring breathing and C–O stretching.^50^ At 1400 cm^–1^, another band is observed and is related to the CH_3_ bending of a methoxyl; at 2700 cm^–1^, we have a symmetric C–H stretching in CH_3_, and the one at 2980 cm^–1^ is the asymmetric one.^50,51^

The band at 1600 cm^–1^ is related to water bending, and those at 3200 and 3600 cm^–1^ are related to water stretching^25,52–54^ and phenloic ν_OH_. The band at 3660 cm^–1^ is related to free OH^–^ ions.^55,56^ The band at 2300 cm^–1^ is related to some CO_2_ generation, and that at 1570 cm^–1^ to carbonyl groups that can be related to S or G rings.^50,51^

Most of these bands are becoming more negative with a potential increment, which means that most are generated at or attracted to the electrode surface. The intensity behavior of the lignin and product related bands is shown in Figure 4.

**Figure 4:**
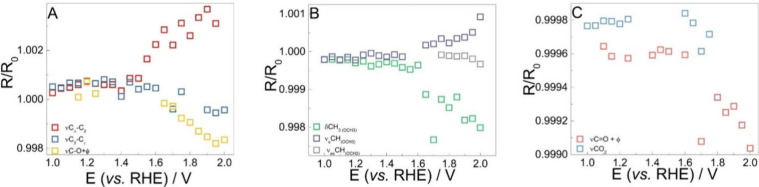
*R*/*R*_0_ vs bias potential obtained for the (A) β-O-4 linkage-related moieties, (B) methoxyl group at the *meta* position, and (C) intermediates/product-related bands.

In Figure 4A, the behavior of three bands is shown, the ones at the lowest wavenumbers. One is becoming more positive, while the other two are negative. The positive one is related to C_α_–C_β_ bond breaking. This attribution comes from the fact that it is at higher wavenumbers than expected for a C–C stretching, indicating a higher polarization, but also it is closer to that observed for C–C stretching of amino acids.^49^ This suggestion will be further discussed by comparison with theoretical calculations. This band increment starts to be expressed at potentials more positive than 1.4 V_RHE_, which is related to the Tafel region observed previously.^6^ This potential seems to be the *E*_onset_ for all band variations in Figure 4.

The decreasing bands in Figure 4A are at 1100 and 1200 cm^–1^. The first one, attributed to the C_β_–C_γ_ bond,^50^ is becoming more negative band probably due to its attraction to the electrode surface after the C_α_–C_β_ bond breaking. The one at 1200 cm^–1^ is related to a ν_C–O_ of an ether, summed with a ring breathing.^50^

Figure 4B presents the bands related to the methoxyl groups in the *meta* position. The δ_CH_3__ at 1400 cm^–1^ is becoming more negative, while the ν_sCH_ at 2700 cm^–1^ is a becoming more positive. This behavior indicates that the monomers are not interacting in parallel to the electrode; they are at least twisted, in a position in which the stretching mode is observed less and the bending mode is observed more. This twisting also makes the ν_sCH_ at 2970 cm^–1^ almost unchanged, until the potential is too positive, and the band becomes negative.

Finally, Figure 4C shows the bands related to the oxidation products. The CO_2_-related band around 2300 cm^–1^ is observed at potentials more positive than 1.6 V_RHE_, as a band that is becoming more negative. This is probably due to fragmentation of smaller structure pieces, as the methoxyl in *meta*, which would justify the increase in selectivity to vanillin, even with this not being the most frequent monomer.^6^ The other band, the carbonyl-related one, is of more interest. Quinones normally present a ν_C=O_ around 1700 cm^–1^.^57^ However, when these structures presented longer resonance structures, this band is shifted to lower wavenumbers, which could even be around 1600 cm^–1^.^58^ Considering this, and the fact that this band starts to be negative even before the ν_C_α_–C_β__ presents any changes (Figure 4A) and is dependent on potential (Figure 4C), this should be related to a charge transfer process that would precede the C_α_–C_β_ bond breaking. In this way, methylene quinone is proposed. This proposition is also in agreement with the well-known band shift of carbonyls when in resonance with the C=C bond.^59^

However, before a mechanism for C_α_–C_β_ breaking is proposed, the interaction between these moieties and the electrode surface needs to be understood. The Stark tuning shift^60–62^ can be of great importance for this analysis, since the band shift with bias potential is evidence of adsorption and the values can be related to the adsorption strength.^25,63,64^ For this analysis, the observed band center was plotted against the applied potential, as shown in Figure 5.

**Figure 5:**
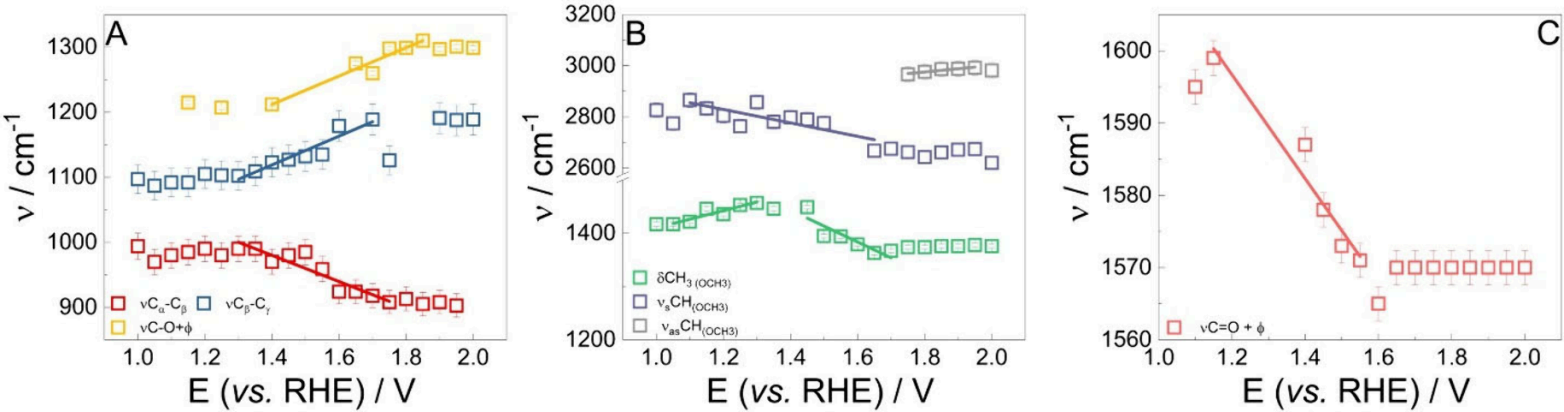
Stark tuning plots (ν vs bias potential) obtained for the (A) β-O-4 linkage, (B) methoxyl group at the *meta* position, and (C) product-related bands.

In Figure 5A, the ν_C_β_–C_γ__ presented a slope of 110 ± 30 cm^–1^ V^–1^, and the coupled vibration ν_C–O_ + φ a slope of 132 ± 30 cm^–1^ V^–1^. The band related to ν_C_α_–C_β__ presents a slope around −162 ± 28 cm^–1^ V^–1^. This is a very strong shift. However, as one can see, the curve is not purely linear; there is a nonlinear factor in it that can be a result of innumerous factors, one being the electric double-layer structure.^65^ The consumed moiety was the only moiety that presented a negative slope. This phenomenon could be related to a decrease in the adsorption strength with the applied potential^63^ or to an increment in the reactivity of such a moiety. To improve our comprehension, theoretical calculations were performed.

The power spectra, generated from the trajectory analysis using TRAVIS, for the physisorbed and chemisorbed lining in CuO (111) are shown in Figure 6A. The main feature in the chemisorbed form is in the OH–C_γ_ moiety. The H is no longer present in this OH group, and a bond now exists between this oxygen and a Cu from the surface. Such reactivity is expected for the selectivity to aldehydes, since some mechanisms propose that the initial oxidation is by C_γ_.^6^ The spectral difference, which better correlates with Figure 3A, is shown in Figure 6B and shows some relevant changes that are highlighted in Figure 6C.

**Figure 6:**
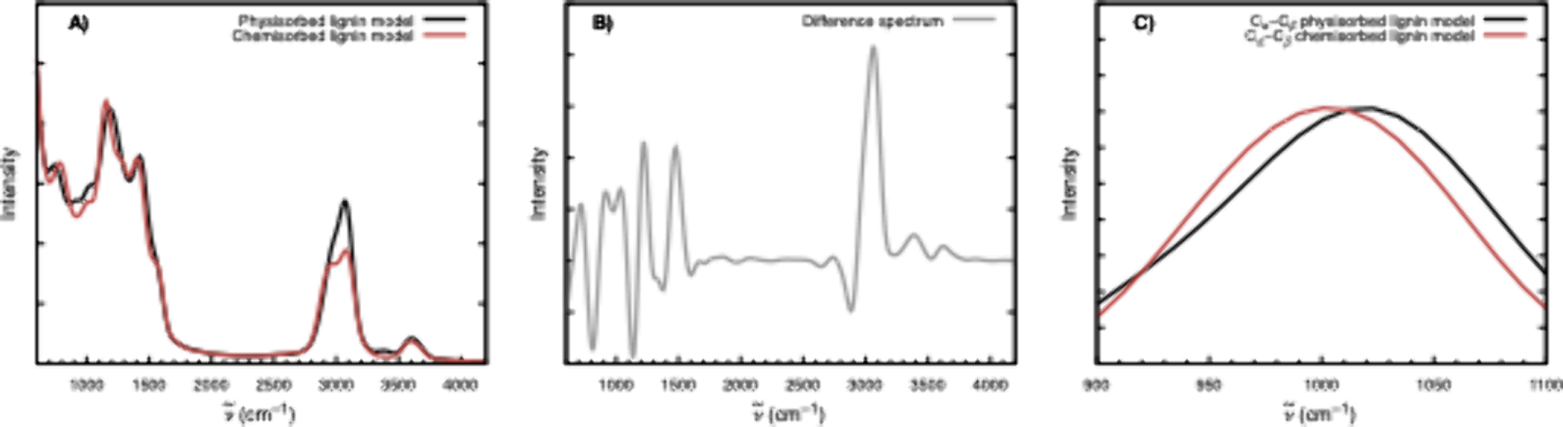
Simulated power spectra: (A) physisorbed and chemisorbed lignin models, (B) difference spectrum between physisorbed and chemisorbed spectra, and (C) contributions of the C_α_ and C_β_ atoms to the calculated power spectrum.

For the ν_C_α_–C_β__ (Figure 6C), the observed maxima are at 1019 cm^–1^ for the physically adsorbed species and 1003 cm^–1^ for the chemically adsorbed species. Following the reasoning that the physically adsorbed species dominates at low potentials while the chemically adsorbed species prevails at higher potentials, we expect that as the potential increases, the signal of this band shifts from 1019 to 1003 cm^–1^. This trend is roughly reflected in the experimental data in Figure 5A (red points). In this way, the negative shift reflects, actually, chemical adsorption and not typical Stark perturbation.

In Figure 5B, two negative Stark shifts are observed, one related to the δ_CH_3__, at potentials more positive than 1.50 V_RHE_, −376 ± 70 cm^–1^ V^–1^, and the ν_sC–H_, −298 ± 50 cm^–1^ V^–1^. The first band at more negative potentials presented a positive shift of around 115 ± 30 cm^–1^ V^–1^. The positive shift suggests that this moiety starts to have a strong interaction between the electrode surface, and afterward, when the band reflectance starts to change, the shift becomes negative. In this potential window, the electrode is expected to present more structural defects, like O vacancies.^6^ The two bands became desorbed at potentials more positive than 1.70 V_RHE_, as they can be seen as stationary. On the other hand, at this same potential, the ν_asC–H_ presents a positive shift of 128 ± 21 cm^–1^ V^–1^. It is interesting to note that, in this case, the positive shifting mode is the only one that presents consumption behavior (Figure 4B).

For the intermediates/product-observed bands (Figure 5C), no shift was observed for the CO_2_-related band. This molecule seems to be generated by oxidation of the adsorbed methoxyl. The quinone band, by its turn, has shown a negative initial slope, −70 ± 10 cm^–1^ V^–1^, which in modulus is close to that observed for CO adsorbed on Pt.^27,60^ At potentials more positive than 1.6 V_RHE_, the band becomes stationary.

Having negative Stark tuning slopes is, at least, unexpected. The theoretical calculations helped to understand what was going on with the C_α_–C_β_ bond but could not fully explain the other negative values. The literature presents some discussions about negative values. It was already observed for CO on Pt,^27,60^ for example. This anomalous behavior was already a stage for large discussions, especially because for CO it starts as a positive slope and when the oxidation starts to be expressive, it becomes negative.

In this potential window, it was shown in the literature that Cu-based materials are affected by a severe morphology modification,^6,66^ which is related to changes in the oxidation state, and all of the following expected changes. This was the reason for the O vacancies observed in this system, for example.^6^ For Pt electrodes, more specifically, for single-crystal Pt electrodes, the oxidation is also related to severe morphological and crystallographic modifications. Recently, Diesen et al. suggested that the anomaly of this negative slope for CO adsorbed on Pt is related to these changes.^67^ They proposed that this would be an artifact related to the consideration of CO adsorption on more than one crystal at the same time. Since δ_CH_3__ (Figure 5B) also presented an initial positive slope and afterward, during the largest area/vacancy population variation,^6^ the slope becomes negative, this can be the reason.

The quinone band is not related to a reactant and is negative even before the largest structural changes. Some works related the Stark shift not to the Stark effect, but to the interaction between the adsorbed species.^68,69^ In most cases, the adsorbed species interact with each other following a Frumkin isotherm model.^70–73^ For most cases, as for CO on Pt, the interaction measured by the heterogeneity factor is related to a repulsion, so to continue to fully cover the surface, more energy and more overpotential need to be supplied to the system. In all of these cases, a positive slope is observed. Therefore, attractive interactions, a negative heterogeneity factor, can be one reason for such behavior.

Based on the experimental evidence shown in Figures 4 and 5 and the simulations presented in Figure 6, the first step is the oxidative adsorption of lignin on the electrode surface. This step generates an adsorbed intermediate (**A**), in which the lignin bonding site is bonded to C_γ_. This intermediate can be reorganized to generate the adsorbed quinone species suggested by an equilibrium (**B**). Further reaction would be due to the oxidation of C_γ_ to carbonyl in a desorbed species (**C**). A retro-aldol step takes place using this desorbed intermediate as a reactant, breaking the C_α_–C_β_ linkage. These steps are consistent with our experimental findings and are summarized in Scheme 3.

**Scheme 3:**
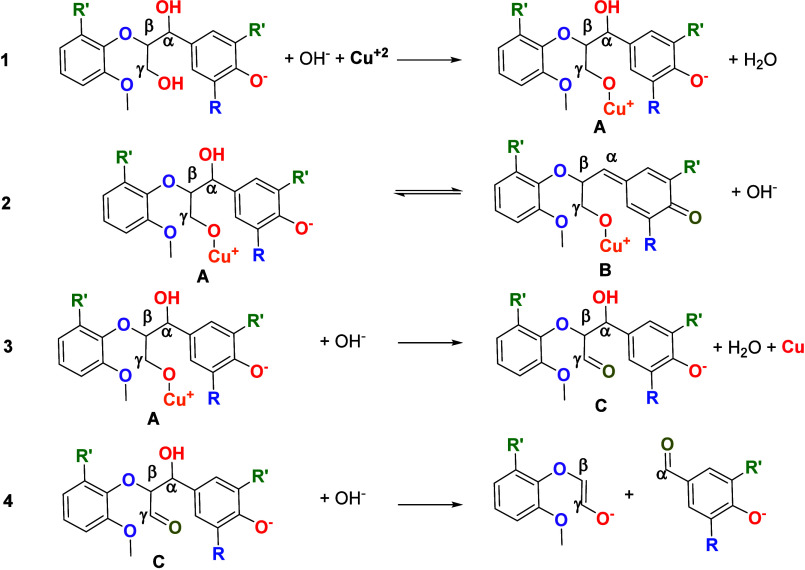
Proposed Mechanism for the Oxidative Breaking of the β-O-4 Linkage

This mechanism presents the aldehyde as a nonadsorbed intermediate, which could explain why no bands related to them were observed. The ν_C=O_ signal could also be in the same region as the quinone band. The data presented in this work could not distinguish between them.

## Conclusions

By a detailed spectroelectrochemical investigation, the lignin and oxidation intermediate-related bands were observed and related to the proposed mechanism for oxidative depolymerization. The lignin–electrode interaction could be observed, and a mechanism containing steps analogous to the chemical oxidation could be proposed but now considering the adsorption of intermediates and the presence of the methylene quinone intermediate. C_α_–C_β_ bond breaking was observed by the reactant behavior of the ν_C_α_–C_β__ signal.

Showing that it is possible to evaluate *in situ* the interaction of complex molecules, such as lignin, and heterogeneous catalysts is an insight for the molecular point of view over (electro)­biorefinery, providing new horizons for these investigations and improving its application for new, renewable, and greener feedstock acquisition.

## Supplementary Material



## Notes

Crystallography investigation by X-ray diffraction of the catalyst and its interpretation, all raw FTIR spectra considered for this work, a brief description of the data treatment, a table containing the statistics of fit quality obtained, and a molecular weight investigation of the isolated lignin by gel permeation chromatography and its interpretation (PDF)

## ∇

Institute of Chemistry, São Paulo State University, Av. Prof. Francisco Degni, 55, Araraquara 14800-900, Brazil

## Abbreviations

H: *p*-coumaryl alcohol
G: coniferyl alcohol
S: sinapyl alcohol
LEOR: lignin electrochemical oxidation reaction
IRRAS: infrared reflection–absorption spectroscopy
RHE: reversible hydrogen electrode
ASE: Atomic Simulation Environment
VDOS: vibrational density of states
