# Supplementary material for: Electrochemical Lignin Oxidation Reaction on CuO: In Situ Spectroelectrochemical Point of View
Source: Langmuir. 2025 Aug 15;41(33):22390–9. doi: 10.1021/acs.langmuir.5c02744 (PMC12392720; doi:10.1021/acs.langmuir.5c02744)
Supplement: Supplementary file 1 [file la5c02744_si_001.pdf]

Support Information to

Electrochemical Lignin Oxidation Reaction on CuO: *in situ* Spectroelectrochemical Point of View

André H. B. Dourado,[1,2]* Matheus Santos,[1] Ana P. de Lima Batista,[3] Antonio G. S. de Oliveira-Filho,[1] Antonio A. S. Curvelo,[1] Hamilton Varela[1]*

[1]São Carlos Institute of Chemistry, University of São Paulo, Av. Trab. Sancarlense, 400, São Carlos, 13566-590 – Brazil

[2(present adress)] Institute of Chemistry, São Paulo State University, Av. Prof. Francisco Degni, 55, Araraquara, 14800-900 – Brazil

[3] Departamento de Química, Grupo Computacional de Catálise e Espectroscopia (GCCE), Universidade Federal de São Carlos (UFSCar), São Carlos, SP 13565-905 - Brazil

*andre.dourado@unesp.br (AHBD)

*hamiltonvarela@usp.br (HV)

This file contains the crystallography investigation by X-ray diffraction of the catalyst, as well as its interpretation, all raw FTIR spectra considered for this work, together with a brief description of the data treatment, and a table containing the statistics of fit quality obtained. Finally, it finishes with the molecular weight investigation of the isolated lignin by gel permeation chromatography and its interpretation.

1. Catalyst structure

For the theoretical calculations, the crystallographic structure of CuO was needed. For that, X-ray diffraction was performed:

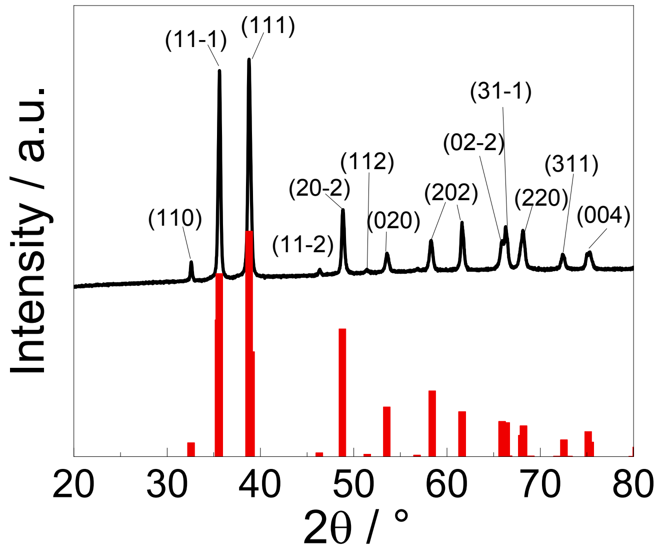

Figure S1. X-ray diffractogram for the CuO powder. The two most intense signals at 35.6 and 38.7º are evidence that the (111) facet is the most present in this polycrystalline material. That is why this facet was selected for the simulations. The signal attribution agrees with the literature[1] available at the ICDS crystallographic database.

2. Spectroscopic data

The main text shows some raw spectra, at different potentials and one example of the data treatment presented. In here, we present all raw spectra from 1.0 V_{RHE} up to 2.0 V_{RHE} every 50 mV.

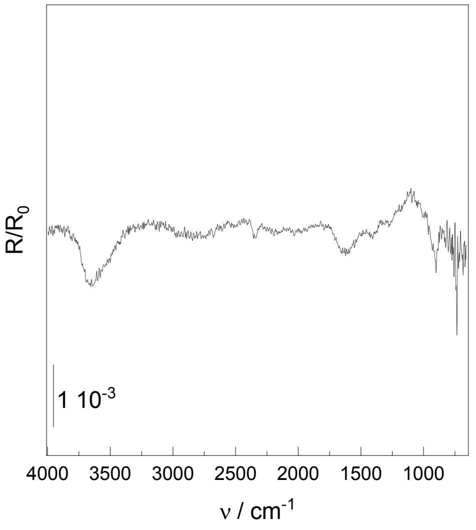

Figure S2. Raw spectrum pleasured at 1.00 V_{RHE}

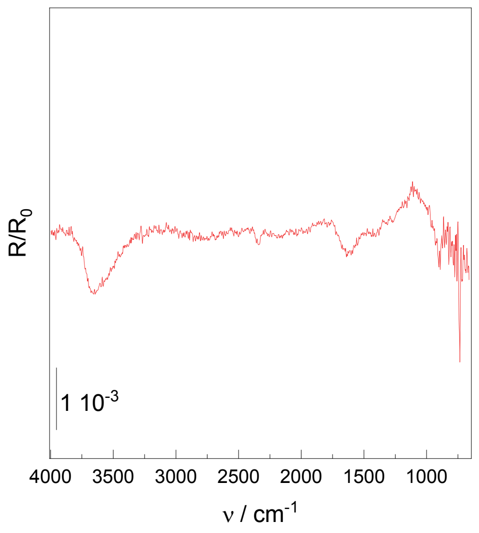

Figure S3. Raw spectrum pleasured at 1.05 V_{RHE}

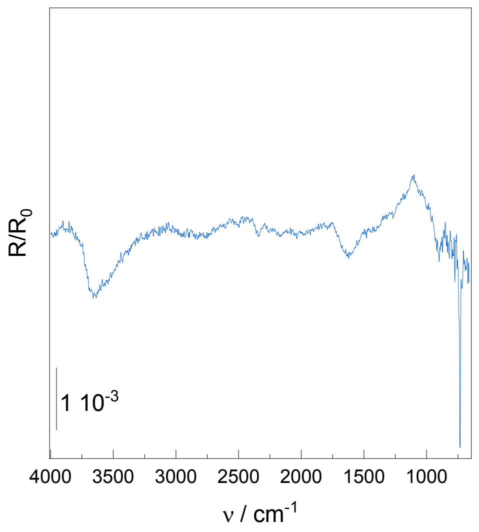

Figure S4. Raw spectrum pleasured at 1.10 V_{RHE}

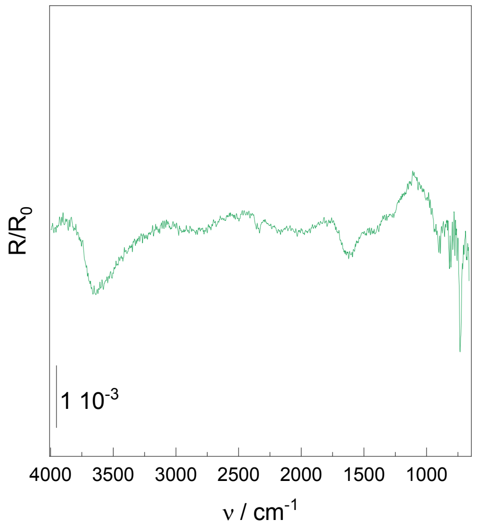

Figure S5. Raw spectrum pleasured at 1.15 V_{RHE}

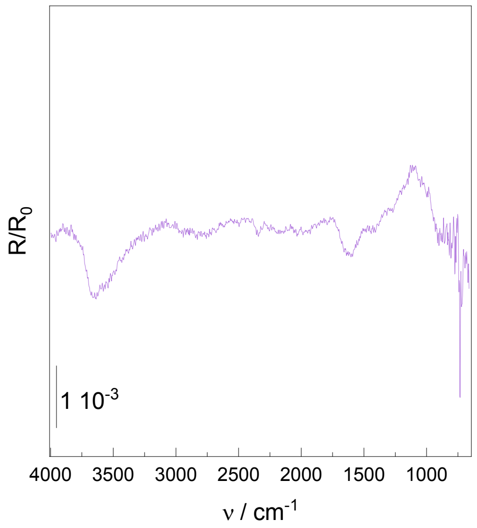

Figure S6. Raw spectrum pleasured at 1.20 V_{RHE}

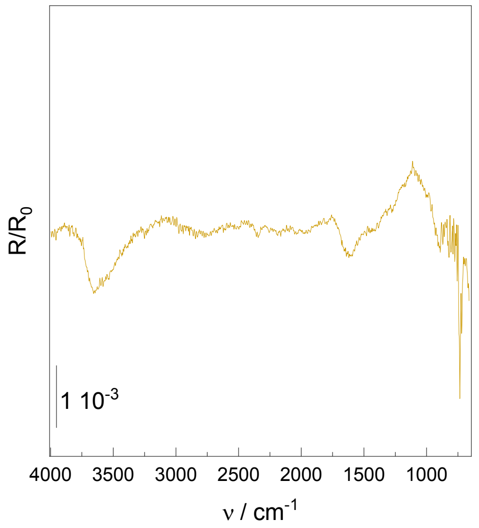

Figure S7. Raw spectrum pleasured at 1.25 V_{RHE}

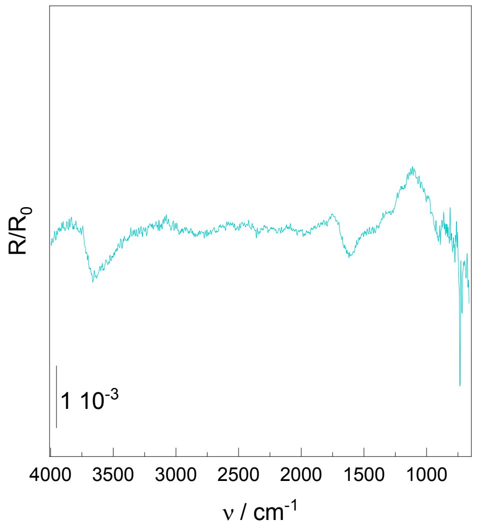

Figure S8. Raw spectrum pleasured at 1.30 V_{RHE}

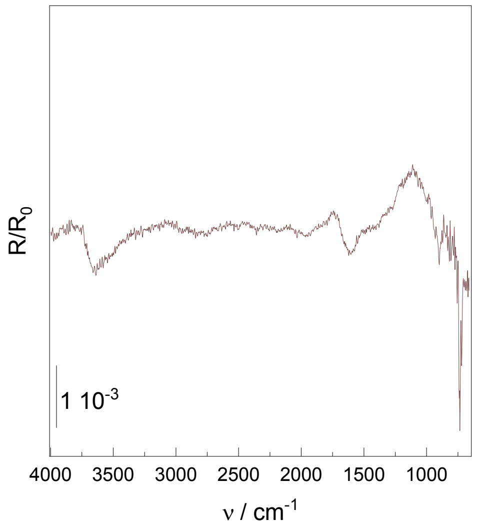

Figure S9. Raw spectrum pleasured at 1.35 V_{RHE}

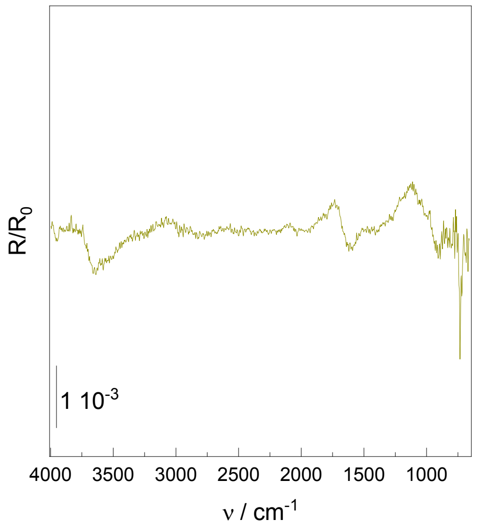

Figure S10. Raw spectrum pleasured at 1.40 V_{RHE}

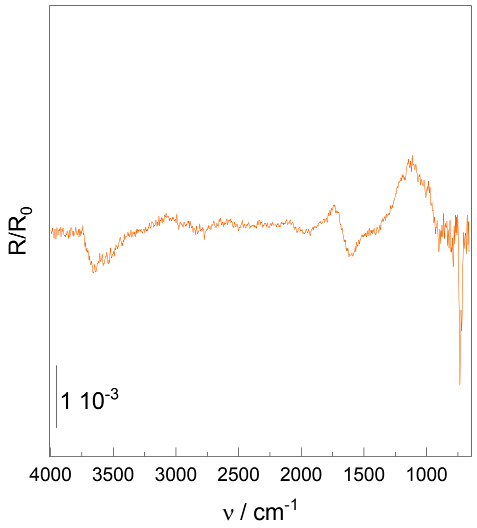

Figure S11. Raw spectrum pleasured at 1.45 V_{RHE}

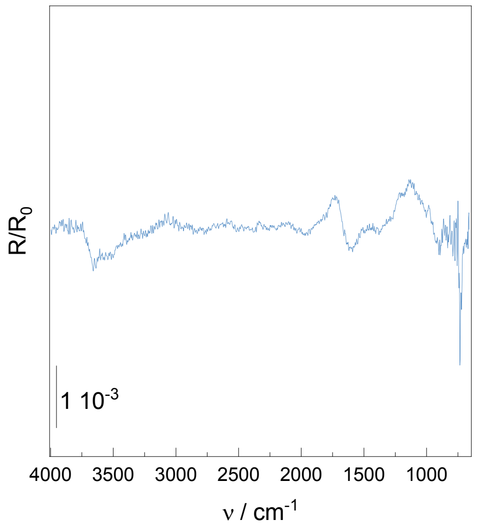

Figure S12. Raw spectrum pleasured at 1.50 V_{RHE}

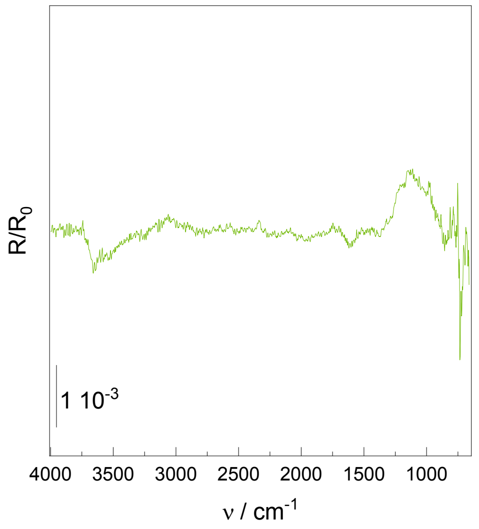

Figure S13. Raw spectrum pleasured at 1.55 V_{RHE}

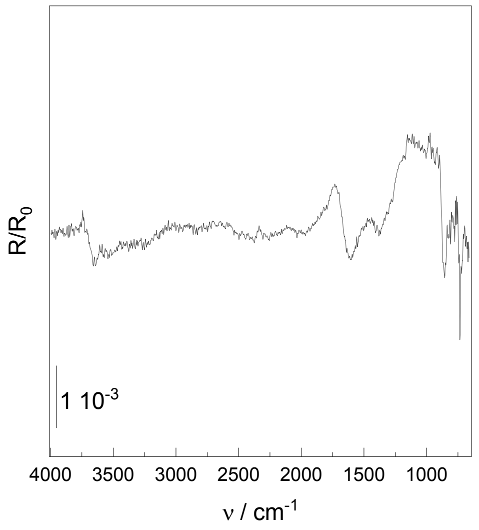

Figure S14. Raw spectrum pleasured at 1.60 V_{RHE}

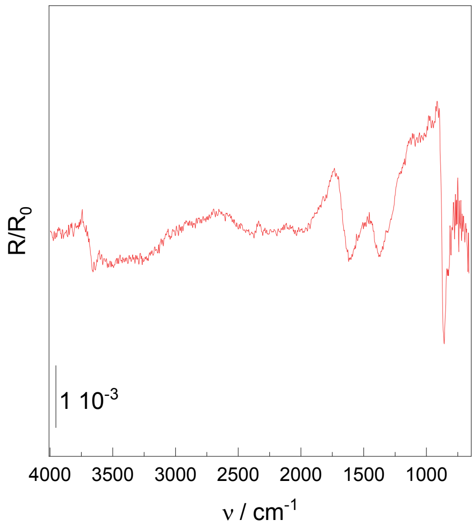

Figure S15. Raw spectrum pleasured at 1.65 V_{RHE}

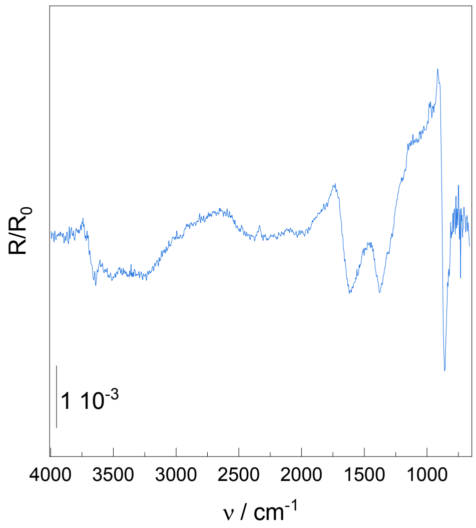

Figure S16. Raw spectrum pleasured at 1.70 V_{RHE}

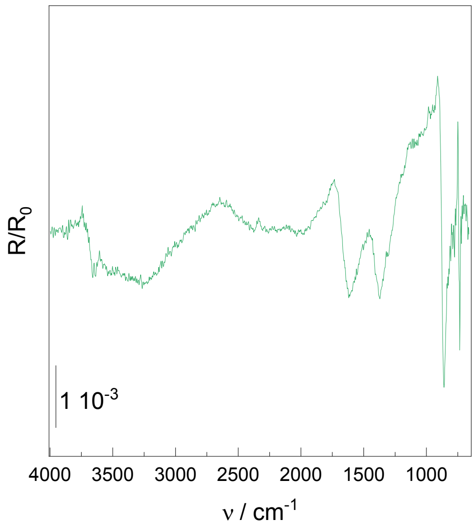

Figure S17. Raw spectrum pleasured at 1.75 V_{RHE}

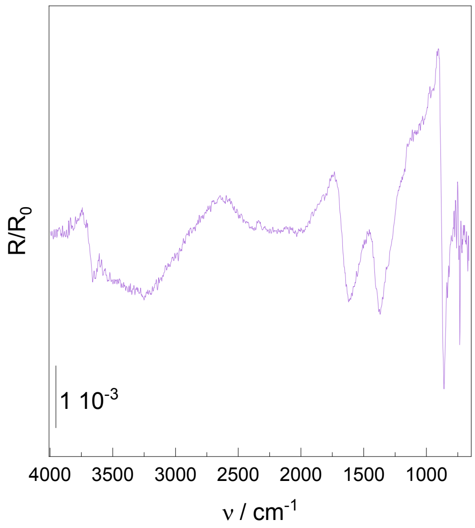

Figure S18. Raw spectrum pleasured at 1.80 V_{RHE}

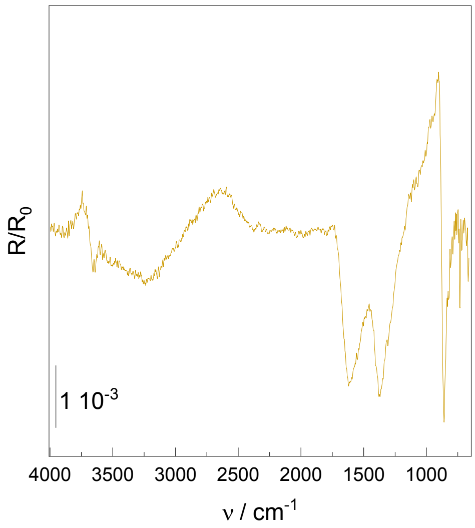

Figure S19. Raw spectrum pleasured at 1.85 V_{RHE}

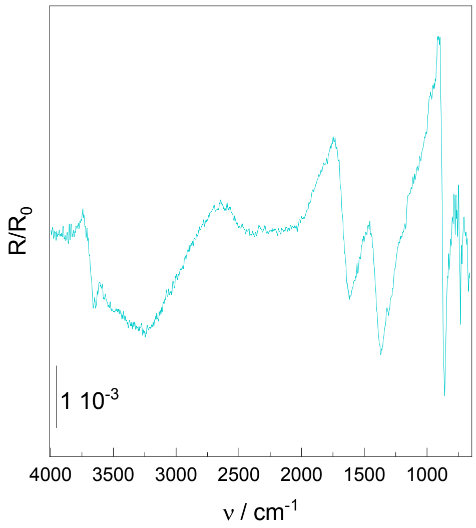

Figure S20. Raw spectrum pleasured at 1.90 V_{RHE}

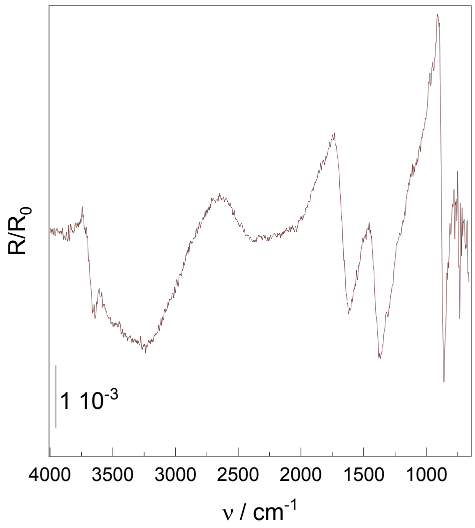

Figure S21. Raw spectrum pleasured at 1.95 V_{RHE}

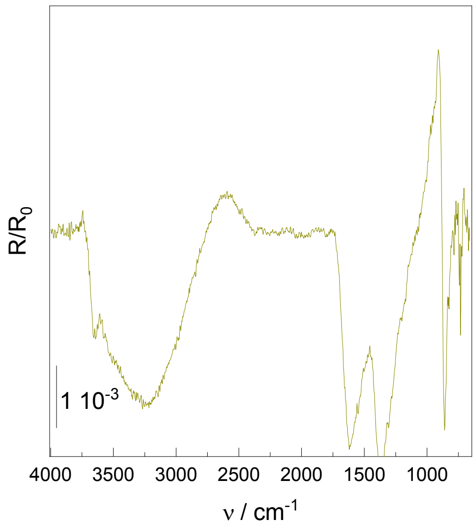

Figure S22. Raw spectrum pleasured at 2.00 V_{RHE}

About data treatment, we used the software OriginPro 2025. There was no water-background correction, no smoothing and the baseline, as can be seen for the raw data, was not corrected strongly. We split each spectrum in two, between 780 and 2100 cm^{-1} and

2200 and 4000 cm^{-1}. Each part was then subjected to the Fit Peaks tool into the Peak Analyzer menu. The baseline, as previously cited, was not strongly corrected, so the connected points were enough. The bands were considered just when their intensity was much higher than the background noise and when the bands were not symmetrical, a second band was added to the adjustment. We considered gaussian shaped bands. The comparison between the raw data and the fitted one can be seen in Figure 3B and 3C, where the experimental measurements are the open squares, the cumulative fitting, the red line, as well as the individual bands presented in different colors. The statistics about the fit quality are presented in Table S1.

Table S1. Quality of spectra adjustment

Applied potential / V	Wavenumber window / cm^{-1}	χ^2
1.00	780 - 2100	4.20E-09
	2200-4000	2.32E-09
1.05	780 - 2100	6.16E-09
	2200-4000	2.11E-09
1.10	780 - 2100	8.09E-09
	2200-4000	4.08E-09
1.20	780 - 2100	1.01E-08
	2200-4000	3.26E-09
1.25	780 - 2100	1.10E-08
	2200-4000	3.10E-09
1.30	780 - 2100	5.43E-09
	2200-4000	3.22E-09
1.35	780 - 2100	1.25E-08
	2200-4000	2.39E-09
1.40	780 - 2100	1.00E-08
	2200-4000	1.99E-09
1.45	780 - 2100	1.50E-08
	2200-4000	3.45E-09
1.50	780 - 2100	1.52E-08
	2200-4000	2.58E-09
1.55	780 - 2100	5.70E-09
	2200-4000	2.46E-09
1.60	780 - 2100	3.25E-08
	2200-4000	5.20E-09
1.65	780 - 2100	3.25E-08
	2200-4000	2.85E-09
1.70	780 - 2100	4.02E-08

	2200-4000	2.46E-09
1.75	780 - 2100	7.38E-08
	2200-4000	2.70E-09
1.80	780 - 2100	1.00E-08
	2200-4000	2.70E-09
1.85	780 - 2100	9.99E-09
	2200-4000	2.78E-09
1.90	780 - 2100	1.00E-08
	2200-4000	3.24E-09
1.95	780 - 2100	1.00E-08
	2200-4000	2.59E-09
2.00	780 - 2100	5.08E-08
	2200-4000	3.19E-09

3. Molecular Weight

For the gel permeation chromatography (GPC) of the Pepper Lignin 5 mg of the sample was dissolved in 2 mL of tetrahydrofuran (THF). The analysis was carried out on a system equipped with three PL_{gel} columns in series (500 Å, 10^3 Å, and 10^4 Å). THF was used as the mobile phase at a flow rate of 1.0 mL min^{-1} and a column temperature of 35 °C. Detection was performed using UV-Vis detector at 254 nm. The weight-average molecular weight (M_w) was estimated by comparison with a calibration curve obtained using polystyrene standards.

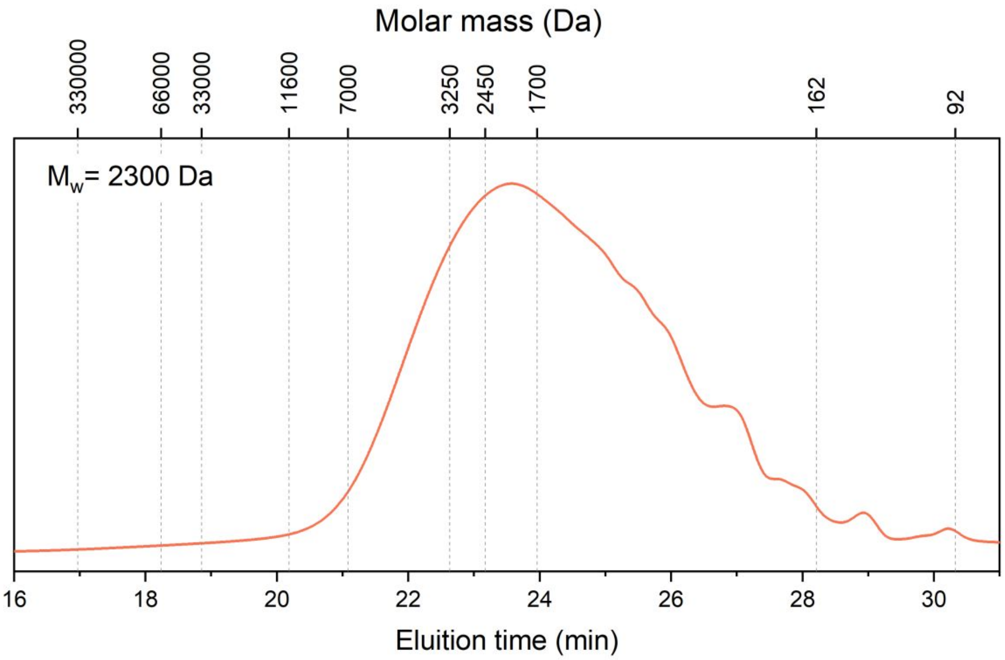

Figure S23. GPC chromatogram of the lignin isolated by the Pepper method from sugarcane bagasse, used in the LEOR experiments.

Pepper lignin presents large molar mass distribution with average M_w, average M_n and dispersity index of 2300 Da, 800 Da, 2.9, respectively. The oxidative depolymerization carried in electrocatalytic reactions led to the formation of monophenols, as identified and quantified by GC analysis. The presence of such compounds in the product of ELOR shows that extensive depolymerization took place. However, comparison between the average M_w its distribution in samples after LEOR was not possible as the reactions were carried on small scale and not enough samples are produced for GPC analysis. Furthermore, as reported in the literature, the M_w of samples isolated after oxidative catalytic depolymerization of in-situ lignin, although isolated from different biomass, presents M_w around 250 Da.[2] Even if the oxidative process carried out was not electrochemical but thermochemical, previous works shows similar reactions pathways and tendencies in the reaction.[3]

References

(1) Åsbrink, S.; Norrby, L. J. A Refinement of the Crystal Structure of Copper(II) Oxide with a Discussion of Some Exceptional e.s.d.'s. *Acta Crystallogr B* **1970**, *26* (1), 8–15. https://doi.org/10.1107/S0567740870001838.

(2) Zhu, Y.; Liao, Y.; Lu, L.; Lv, W.; Liu, J.; Song, X.; Wu, J.; Li, L.; Wang, C.; Ma, L.; Sels, B. F. Oxidative Catalytic Fractionation of Lignocellulose to High-Yield Aromatic Aldehyde Monomers and Pure Cellulose. *ACS Catal* **2023**, 7929–7941. https://doi.org/10.1021/acscatal.3c01309.

(3) Dourado, A. H. B.; Santos, M.; Curvelo, A. A. S.; Varela, H. CuO as (Electro)Catalyst for Lignin Valorization. *Appl Catal A Gen* **2024**, *671*, 119583. https://doi.org/10.1016/j.apcata.2024.119583.
